# Incidence and microbiology of post-operative infections after radical cystectomy and ureteral stent removal; a retrospective cohort study

**DOI:** 10.1186/s12879-019-3932-4

**Published:** 2019-04-03

**Authors:** E. Kolwijck, A. E. M. Seegers, S. C. M. Tops, A. G. van der Heijden, J. P. M. Sedelaar, J. ten Oever

**Affiliations:** 1 Radboud center for infectious diseases, Radboud university medical center, P.O. Box 9101, 6500 HB Nijmegen, the Netherlands; 2 Department of medical microbiology, Radboud university medical center, P.O. Box 9101, 6500 HB Nijmegen, the Netherlands; 3 Department of internal medicine, Radboud university medical center, Nijmegen, the Netherlands; 4 Department of urology, Radboud university medical center, Nijmegen, the Netherlands

**Keywords:** Antibiotic prophylaxis, Post-operative infection, Ureteral stent, Radical cystectomy, Microbiology

## Abstract

**Background:**

Post-operative infections are frequent after radical cystectomy with urinary diversion surgery (UDS). Reduction of post-operative infections necessitates appropriate peri-operative antimicrobial prophylaxis targeting causative bacteria. We assessed the incidence and microbiology of infections in the 30-day post-operative period after UDS and investigated effectiveness of the currently used peri-operative antibacterial prophylaxis.

**Methods:**

Retrospective cohort study of all patients undergoing UDS in a tertiary university medical center from January 2014 until September 2016. Antibiotic prophylaxis consisted of cefazolin plus metronidazol according to the Dutch national guideline. Primary outcome was the incidence of post-operative infections within 30 days. Risk factors for post-operative infections and antimicrobial susceptibility profiles of cultured bacteria were also assessed.

**Results:**

147 patients were included. 69 patients (46.9%) had 82 post-operative infections, 27 of which were patients with bacteremia (18.4%). Highest incidence of infections was on day 4–5 and on day 8–10 postoperatively. The second peak was associated with ureteral stent removal. 4.8% of 147 study patients developed bacteremia 24 h after stent removal, which counted for 25.9% of all bacteremia episodes found in this study. Enterobacteriaceae were cultured in 67.9% of blood cultures and were only highly susceptible to ciprofloxacine, piperacillin-tazobactam (90%), meropenem and gentamicin (100%). Multivariate logistic regression analysis showed orthotopic Hautmann neobladder to be associated with increased infections complications: odds ratio 4.1 (95% confidence interval 1.6–10.5), *p* = 0.03.

**Conclusions:**

The incidence of infections after radical cystectomy is high and particularly ureteral stent removal was associated with both bacteremia and complicated urinary tract infections. Based on the results of this study, antibiotic prophylaxis might need to be broadened for patients undergoing radical cystectomy. Further research is required to investigate whether current guidelines need to be altered concerning administration of antibiotic prophylaxis just before stent removal.

**Electronic supplementary material:**

The online version of this article (10.1186/s12879-019-3932-4) contains supplementary material, which is available to authorized users.

## Background

Ileal conduit (IC) and orthotopic Hautmann Neobladder (NB) are two types of urinary diversion surgery (UDS) that are frequently performed after radical cystectomy. In both types of UDS, the ureters are attached to the IC or NB using temporal ureteral stents to assure patency and prevent stenosis while healing. The UDS procedure is associated with increased risk of bacteremia, complicated urinary tract infection (cUTI) and surgical site infection (SSI) [1]. The first 30 days, infections contribute significantly to post-operative morbidity [2, 3], which can be explained by multiple comorbidities as well as by the complex surgery that involves attaching part of the colonized gastro-intestinal tract to the sterile urinary tract [4].

Up until now, several studies focused on the incidence of early (< 30 days) post-operative infections [4–11]. Overall infectious complication rates in these studies ranged from 10 to 41% [7, 9, 11]. Of these infectious complications, bacteremia was reported in 5 to 17% of the patients [9–11], cUTI was reported in 4 to 36% of the patients [5, 9–11] and SSI was reported in 8 to 33% of patients [4, 6–9, 11] after UDS, even when peri-operative antimicrobial prophylaxis was used. Although some of these studies reported on the causative bacteria [4, 5, 8–10], we found that only a few studies partially provided information on antimicrobial susceptibility of the cultured bacteria [4, 8, 9]. Particularly in the era of increasing antimicrobial resistance, susceptibility profiles provide an important pillar in the correct use of antimicrobial prophylaxis [7, 12]. The recommended guideline-based peri-operative antimicrobial prophylaxis for UDS in the Netherlands consists of cefazolin plus metronidazol [13]. Cefazolin is one of the most prescribed (40%) prophylactic antibiotics for radical cystectomy in the United States as well [14]. However, the same large cohort study showed that a penicillin-based regimen with a beta-lactamase inhibitor was associated with the least post-operative complications [14], suggesting increased gram-negative coverage is necessary in the era of increasing resistance.

Guidelines [13, 15] advise not to extend the prophylaxis beyond 24 h after surgery, which is further supported by a recent study [14]. Interestingly, none of the guidelines specifically recommends on the need and type of antibiotic prophylaxis during ureteral stent removal after UDS [15–17]. Ureteral stent removal is typically performed at least a week after UDS and involves manipulation in a non-sterile environment attached to the urological tract, which might predispose to infections, similar to manipulation of an obstructed common bile duct [18]. European [15] and American guidelines [16] do recommend antibiotic prophylaxis for patients undergoing cystourethroscopy (low quality of evidence) based on a few studies that assessed effectiveness of prophylactic antibiotics during upper urinary tract stone treatment, but this might not be representative to ureteral stent removal after UDS.

Given these shortcomings, the aim of this study was to ascertain the efficacy of antimicrobial prophylaxis by investigating the incidence and antibiotic susceptibility of causative micro-organisms of post-operative infections after IC or NB urinary diversion surgery. Additionally, we specifically investigated the incidence and microbiology of infections after ureteral stent removal.

## Methods

We conducted a retrospective cohort study in the Radboud university medical center, a university hospital in the Netherlands performing 80–100 radical cystectomies per year, by using a prospective database of patients undergoing radical cystectomy followed by UDS.

Inclusion criteria were patients aged ≥18 years and undergoing either IC or NB diversion between January 2014 and September 2016 in the Radboud university medical center. Exclusion criteria were an active pre-operative infection, defined as the presence of signs of overt infection, or the prescription of a therapeutic course of antibiotics.

Preparation of patients undergoing UDS consisted of bowel preparation using an osmotic laxative and enemas starting one day prior to surgery. Antibiotic prophylaxis protocol according to the Dutch national guideline consisted of one dose of cefazolin 1000 mg and metronidazole 500 mg administered intravenously prior to incision [17]. In the Dutch guideline no antibiotic prophylaxis is recommended during ureteral stent removal, also no prolonged post-operative prophylaxis is recommended [17]. For a cefazolin dose to be scored as optimal guideline-adherent in our regression analyses, a repeat administration was required in case of prolonged surgery (> 4 h), heavy blood loss (> 2 L), and a double dose in case of a body mass index (BMI) > 35 kg/m^2^ or bodyweight > 120 kg [19]. Removal of the ureteral stents was generally performed on two consecutive days, on day 7 and 8 in case of IC and on day 9 and 10 in case of NB after radical cystectomy.

Data of interest were extracted from the electronic medical record (EPIC) and entered in CastorEDC, a licensed online data collection directory for medical research. The following patient characteristics were collected: sex, age, length, weight, body mass index, American Society of Anesthesiologists (ASA) score, Charlson Comorbidity Index (CCI) score, indication for surgery and T-staging in the case of bladder cancer, previous chemotherapy, and carrier state of a multidrug resistant micro-organism (MDRO) (methicillin-resistant *Staphylococcus aureus*, vancomycin-resistant enterococci, bacteria containing plasmid-mediated AmpC beta-lactamases, extended-spectrum beta-lacamase producing Enterobacteriaceae, or carbapenemase-producing Enterobacteriaceae). Peroperative variables that were collected included duration of surgery, blood loss, timing and dosage of antibiotic prophylaxis and presence of nephrostomy or double-J catheters. Post-operative variables that were collected were (timing of) infections, length of stay and mortality. Table 1 shows the definitions used to classify infections.

**Table 1:** Definitions used for post-operative infectious complications

Complicated urinary tract infection	Temperature ≥ 38.5 °C and a positive urine culture (≥10^5^ CFU/ml and leucocytes in gram stain) without a positive blood culture or SSI
Surgical site infections	CDC criteria [20]
Bacteremia	Temperature ≥ 38.5 °C and a positive blood culture with or without a positive urine culture

The primary outcome measurement was the incidence of post-operative infections either microbiologically confirmed (bacteremia and cUTI) or clinically diagnosed (surgical site infection [SSI] [20]) within 30 days after radical cystectomy (Table 1). These infections were chosen since these can be prevented with preoperative antibiotic prophylaxis. A positive urine culture without concomitant bacteremia in a febrile patient shortly after UDS not necessarily indicates a cUTI, but excluding these patients would underestimate the incidence. Furthermore, the new urinary reservoir is the main portal of entry and the susceptibility of the residing bacteria should guide prophylaxis and empiric treatment. Patients with both a positive blood culture and a positive urine culture taken on the same day (± 1 day) were scored as “bacteremia” and not as “cUTI”. Secondary outcome measurements were timing of these infections with a specific interest in the relationship with ureteral stent removal, the susceptibility profiles of the causative microorganisms, and risk factors for infections within 30 days.

Identification and antimicrobial susceptibility test of the bacteria isolated from post-operative urine and blood cultures were collected from the laboratory information management system (Glims, MIPS, Gent, Belgium). Identification of the bacteria was performed by MALDI-TOF MS (MALDI Biotyper, Bruker, Daltonics, Bremen, Germany). Coagulase-negative staphylococci and viridans streptococci were considered not clinically relevant and were excluded from the analysis. Antimicrobial susceptibility testing was performed with Phoenix (BD Bioscience, Erembodegem, Belgium) and E-test (BioMérieux, AB Biodisk, Solna, Sweden) in accordance with European Committee on Antimicrobial susceptibility testing (EUCAST) methodology. Minimal inhibitory concentration (MIC) values were interpreted and categorized (S, I, and R) according to EUCAST clinical breakpoint table version 7.1. Antimicrobial susceptibility test results for Enterobacteriaceae included amoxicillin (AMX), amoxicillin-clavulanic acid (AMC), piperacillin-tazobactam (PTZ), ceftriaxone (CRO), ceftazidime (CAZ), ciprofloxacin (CIP), trimethoprim-sulfamethoxazole (SXT), meropenem (MEM), and gentamicin (CN). Susceptibility to cefazolin was not assessed since antimicrobial susceptibility EUCAST breakpoints for cefazolin for Enterobacteriaceae do not exist. Susceptibility testing for enterococci included AMX and vancomycin (VAN). Susceptibility testing for staphylococci included cefoxitin (screening test for methicillin susceptibility), and for *Pseudomonas aeruginosa* CIP, CAZ, CN, and MEM. In the analysis of susceptibility profiles we selected the first isolate per species per patient to avoid bias due to multiple testing. In case more than one bacterial species was isolated from a culture, all uropathogens with a unique resistance pattern were included.

Statistical analysis was performed using SPSS 22.0. Continuous variables were analyzed using students t-test in case of normal distribution, and non-parametric tests in case of non-normal distribution. Categorical variables were analyzed using Chi-Square or Fisher’s exact test. We performed multivariate logistic regression analysis to test for risk factors for infection. Prognostic factors associated with a *p*-value of < 0.20 in univariate analysis were included in multivariate analysis. A 2-sided *p*-value of ≤0.05 was considered statistically significant.

The regional institutional review board approved this study and waived the requirement to obtain informed consent.

## Results

### Patient characteristics

Of the 200 patients who underwent radical cystectomy, 36 did not receive surgery at our institution. Of the 164 remaining patients, 17 patients had an active infection at time of surgery and were excluded. The remaining 147 patients were included in our study. A total of 120 patients underwent IC diversion (82%), while 27 (18%) received a NB. Patients that received a NB were younger than those who received an IC diversion (mean age 57.9 [SD 9.55] vs 67.0 [SD 10.1] years, respectively; *p* = 0.004) and had a different distribution of ASA scores (1 [IQR 1–1] vs 1 [IQR 1–2]; *p* = 0.047). Table 2 shows the demographic and clinical characteristics of the included patients.

**Table 2:** Demographic and clinical characteristics of the 147 included patients

Variable	*n (%) or mean ± SD*
Sex, males	112 (76.2)
Age, years	65.4 (10.6)
BMI, kg/m^2^	26.4 (4.1)
(Previous) smoker	68 (46.3)
Hypertension	79 (53.7)
Diabetes mellitus	16 (10.9)
Nephrostomy catheter	14 (10.1)
ASA class	
ASA class 1	20 (13.6)
ASA class 2	91 (61.9)
ASA class 3	36 (24.5)
Indication	
Bladder cancer	123 (81.6)
Stage T ≥ T2	73 (59.3)
Other malignancy	13 (8.8)
Functional	8 (5.4)
Other	3 (2.0)
Colonization with MDRO	7 (4.8)
Guideline-accordant prophylaxis	116 (78.9)
Correct choice	134 (91.2)
Correct dose	124 (92.5)
Administration < 60 min prior to incision	142 (91.2)
Type of diversion	
Ileal conduit	120 (81.6)
Hautmann neobladder	27 (18.4)
Duration surgery, minutes	216 (75)
Blood loss, ml	1031 (700)

Abbreviations: *ASA* American Society of Anesthesiologists, *BMI* body mass index, *IQR* interquartile range, *MDRO* multidrug resistant microorganism, *SD* standard deviation

### Incidence of post-operative infections

**Fig. 1:**
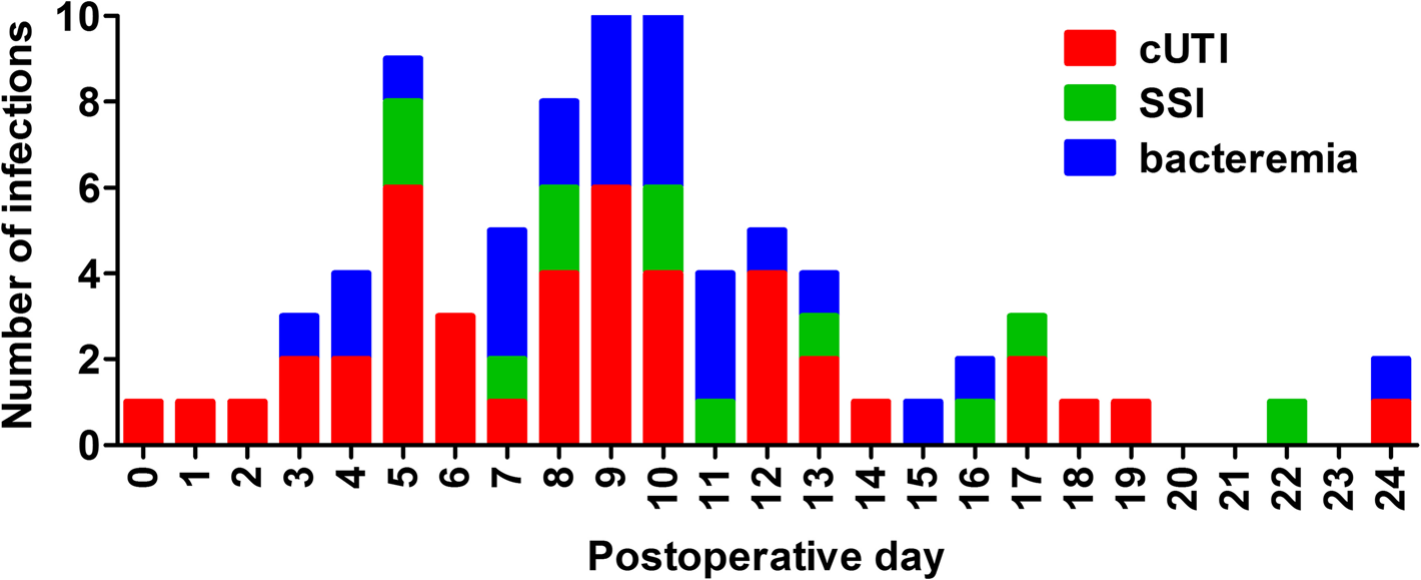
Incidence and distribution in time of the post-operative infections

During the 30-day post-operative period 82 post-operative infections occurred in 69 of 147 patients (46.9%) (Fig. 1). We found 27 episodes of bacteremia (in 27 patients [32.9%]), 43 complicated UTIs (in 41 patients [52.4%], and 12 SSIs (in 11 patients [14.6%]). Twelve patients had more than one infection during the study period. The incidence of post-operative infections peaked on day 4–5 and day 8–10 post-operatively (Fig. 1). The highest incidence of bacteremia was on day 7–10, whereas SSIs only occurred from day 5 onwards and were evenly distributed during the 30-day follow-up period.

### Bacteremia

**Fig. 2:**
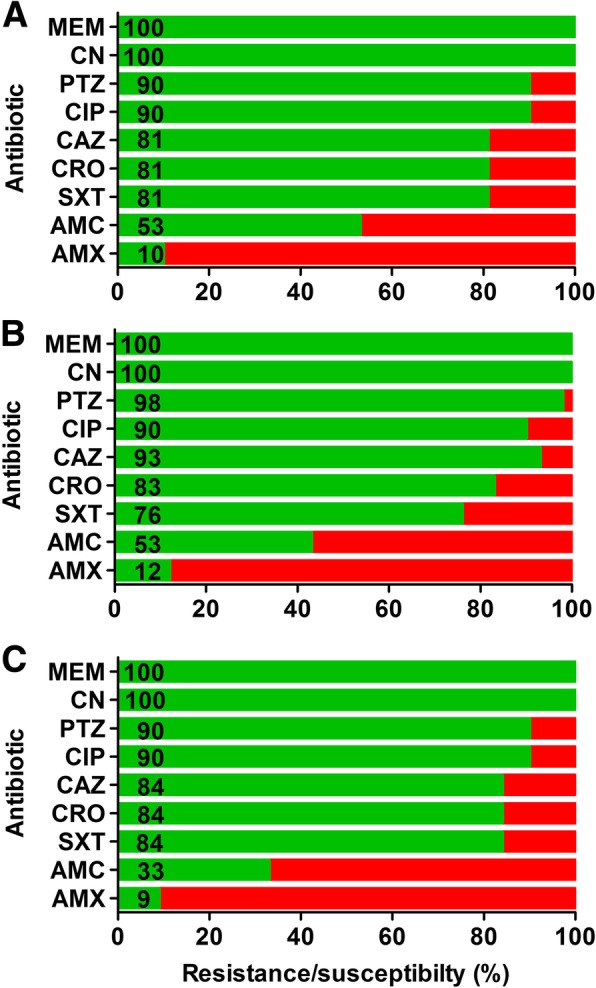
Susceptibility patterns of Enterobacteriaceae and *P.aeruginosa* isolated from (**a**) blood (**b**) urine, and from (**c**) urine in patients with a complicated urinary tract infection 24 h within stent removal. Susceptibility is shown in green, resistance in red. Number in the green bars represents susceptibility percentage

Twenty-seven of 147 patients had bacteremia during the 30-day post-operative period (18.4%). Of 27 patients with positive blood cultures, in 17 patients both the blood culture and urine culture contained identical bacteria, in 7 patients no urine cultures were performed, and in 3 patients the bacteria found in the blood culture differed from the bacteria found in the urine culture. The blood cultures performed grew a total of 28 bacteria (Table 3). Enterobacteriaceae represented the largest group (67.9%), followed by enterococci (18%), *S. aureus* (11%) and *B. fragilis* (4%). In one patient, the blood culture contained both *Klebsiella pneumoniae* and *Proteus mirabilis.* Susceptibility profiles of the Enterobacteriaceae plus *P. aeruginosa* are shown in Fig. 2a. Enterococci were susceptible to AMX in 80% of isolates and susceptibility to VAN was 100%. All *S. aureus* isolates were methicillin susceptible.

**Table 3:** Urine and blood culture isolates from patients with post-operative infections

Bacteria	Urine culture isolates, *n* (%)	Blood culture isolates, *n* (%)
Enterobacteriaceae	41 (62.1)	19 (67.9)
*Escherichia coli*	14 (34.2)	8 (42.1)
*Klebsiella pneumoniae*	9 (22.0)	5 (26.3)
*Enterobacter spp.*	13 (31.7)	4 (21.0)
*Proteus mirabilis*	0 (0)	1 (5.3)
*Serratia marcescens*	1 (2.4)	1(5.3)
*Morganella morganii*	1 (2.4)	0
*Citrobacter spp.*	2 (4.9)	0
*Hafnia alvei*	1 (2.4)	0
*Pseudomonas aeruginosa*	3 (4.6)	0
Enterococci	16 (24.2)	5 (17.9)
*Staphylococcus aureus*	6 (9.1)	3 (10.7)
*Bacteroides fragilis*	0	1 (3.5)
Total	66 (100)	28 (100)

### Complicated UTI

Forty-one of 147 patients had at least one episode of cUTI during the 30-day post-operative period (27.9%). The 43 urine cultures of these 41 patients grew 66 micro-organisms (range of cultured bacteria within 1 culture: 1–3), of which 41 (62.1%) were Enterobacteriaceae (Table 3). The susceptibility profiles of the Enterobacteriaceae plus *P. aeruginosa* are shown in Fig. 2b. Enterococci were cultured in 16 (24.2%) of 66 isolates and 75% of the isolates were susceptible to AMX and 100% to VAN. *S. aureus* formed 9.1% of cultured bacteria and 100% were methicillin susceptible.

### Infections after ureteral stent removal

In the 24 h after ureteral stent removal, which was generally performed on post-operative day 7 through 10 (85.7%), 7 patients developed a bacteremia. This was 25.9% of all patients with a bacteremia found in this study. Six of 7 blood cultures (85.7%) grew Enterobacteriaceae, 1 blood culture grew *B. fragilis* (Table 4). In addition, 16 patients developed a complicated UTI within 24 h after ureteral stent removal. This was 37.2% of all episodes of cUTI found in this study. The 16 positive urine cultures grew a total of 24 micro-organisms (16 [66.7%] Enterobacteriaceae, 5 [20.8%] enterococci, 2 [8.3%] were *S. aureus* and 1 [4.2%] *P. aeruginosa*) (Table 4). Susceptibilities of the Enterobacteriaceae plus *Pseudomona*s from both urine and blood culture isolates are shown in Fig. 2c.

**Table 4:** Urine and blood culture isolates within 24 h after ureteral stent removal

Bacteria	Urine culture isolates, *n* (%)	Blood culture isolates, *n* (%)
Enterobacteriaceae	16(66.7)	6 (85.7)
*Escherichia coli*	4(25.0)	3(50.0)
*Klebsiella pneumoniae*	2(12.5)	1 (16.7)
*Enterobacter spp.*	8(50.0)	2(33.3)
*Proteus mirabilis*	0	0
*Serratia marcescens*	0	0
*Morganella morganii*	1(6.25)	0
*Citrobacter spp.*	1(6.25)	0
*Pseudomonas aeruginosa*	1(4.2)	0
Enterococci	5(20.8)	0
*Staphylococcus aureus*	2(8.3)	0
*Bacteroides fragilis*	0	1 (14.3)
Total	24 (100)	7 (100)

### Outcome and risk factors for post-operative infections

Six patients (4.1%) required surgical treatment, and 8 patients (5.4%) were admitted to the ICU as a result of the infection. The median duration of admission was 14 days (IQR 5 days) and the 30-day mortality rate was 1.4% (*n* = 2), one of whom died of sepsis. In univariate analysis, type of diversion (NB) and MDRO colonization prior to surgery were associated with post-operative infections (Additional file 1: Table S1). Multivariate logistic regression analysis showed only NB to be associated with increased infections complications: odds ratio 4.1 (95% confidence interval 1.6–10.5), *p* = 0.03.

## Discussion

This study showed a high incidence of post-operative infections after radical cystectomy and urinary diversion surgery. Post-operative infections peaked at day 4–5. Furthermore, stent removal (around day 7–10) was associated with the occurrence of bacteremia and cUTI. The cultured gram-negative bacteria in urine and blood samples largely showed an inferred resistance to the used antibiotic prophylaxis regimen consisting of cefazolin. These findings question the timing and choice of cefazolin, currently the most used agent, as surgical antibiotic prophylaxis in radical cystectomy [13–16].

The assessment of etiology and antibiotic susceptibility of post-operative infections characteristics in this study enabled us to broaden the spectrum of antibiotic prophylaxis for radical cystectomy in our hospital to piperacillin/tazobactam and also administer empirical antibiotic prophylaxis just before removal of the ureteral stents. A detailed analysis of the etiology and antibiotic susceptibility of urine and blood isolates does highlight the importance of selecting prophylaxis using the local resistance data. Therefore, this study is an illustrative example that a local guideline should be adapted to local epidemiology of resistance [12, 21]. Another strength of this study is the involvement of stakeholders, as recommended by The Appraisal of Guidelines for REsearch & Evaluation (AGREE) Instrument, a methodological strategy for the development of guidelines [22]. As professional taking care of the target population, the urologist noticed the high incidence of post-operative infections and also hypothesized the relationship with stent removal. Subsequently, the collaboration with a clinical microbiologist and infectious disease specialist enabled the current study and the development of a new guideline. Importantly, the involvement of the prescribing professional, i.e. the urologist, leads to an increased confidence in and facilitates a better implementation of and adherence to a new guideline [22].

Several studies assessed the risk of early post-operative infections after radical cystectomy, showing high incidences but large differences in reported post-operative infection rates. [4–11] In our study, bacteremia was found in 18%, SSI in 8.2% and cUTI in 28% of patients. Rates of early post-operative bacteremia, SSI and UTI (< 30 days) in other studies varied between 5 to 17%, 8 to 33% and 4 to 36%, respectively. [5, 9–11] One explanation for the large differences in reported incidences of post-operative infections after radical cystectomy might be the different types of antibiotic prophylaxis regimens used in different studies. Pariser et al. showed that type of peri-operative antibiotic prophylaxis regimen might have significant effect on infection outcomes after radical cystectomy [7], which was also found in two other studies that analyzed post-operative infections within 90 days after UDS [14, 23]. A recent cohort study in 8351 patients from 353 hospitals in the United States showed that 579 unique peri-operative antibiotic prophylaxis regimens were used. Only 28% of patients received prophylaxis according to the guideline [14]. Also, the diagnosis of cUTI in patients with urinary diversion is complicated by its vague presentation and lack of a standardized definition in the literature. Even though in our study we only diagnosed cUTI when fever (≥ 38.5 °C) could not be explained by SSI or bacteremia (and urine culture showed ≥10^5^ CFU/ml bacteria), we are aware that we might have over reported the amount of cUTI in this study as fever and bacteriuria are common after UDS surgery.

This study particularly focused on the temporal relationship of post-operative infections and the removal of the ureteral stents. To the best of our knowledge, this is the first study that assessed the etiology and antimicrobial susceptibility of bacteria cultured from blood and urine within 24 h after stent removal. We found that 26% of all episodes of bacteremia and 37% of all episodes of cUTI occurred within 24 h after ureteral stent removal. Current guidelines do not recommend on the need and type of antibiotic prophylaxis during ureteral stent removal after UDS [13, 15, 16]. Most probably because the temporal relationship between infection and ureteral stent removal has not been investigated thoroughly, as we could only find two previous studies on this topic [10, 24]. Recently, Werntz et al. analyzed the difference in post-operative infectious complications between 42 patients that received peri-operative and 42 patients that received peri-operative plus extended (30 days) antibiotic prophylaxis after UDS. [10] They found that 30% of patients that received peri-operative prophylaxis only developed a cUTI one day after ureteral stent removal compared to none of the patients in the group that received extended prophylaxis. In a retrospective study performed by Hashimoto et al., prophylactic administration of antibiotics just before stent removal significantly reduced the incidence of febrile events after removal of ureteral stents [24]. Given the high incidence of bacteremia and cUTI within 24 h after stent removal in our study, we believe that, based on the results of this study, antibiotic prophylaxis during ureteral stent removal might reduce post-operative infections after UDS. We encourage administration of a single dose of prophylaxis just before ureteral stent removal rather than extended post-operative antimicrobial prophylaxis, which was administered by Werntz et al. in their study, as this may lead to the development of antimicrobial resistance. [13, 16] However, due to the retrospective and single center design of our study, we feel these findings need to be confirmed in a future prospective randomised clinical trial.

This study has several limitations. The variables of interest were collected retrospectively, which brings along the risk of missing data. However, the outcome parameters are recorded in our EMR and laboratory systems as discrete variables that can be retrieved easily, reducing the risk. Whether a patient is colonized with MDRO was not systematically assessed prior to the operation in most patients, limiting the possibility to draw a conclusion on tailoring prophylaxis to carrier state or structural screening for carrier state of MDRO. Another limitation is that this is a single center study in a country with (relatively) low-level resistance of Enterobacteriaceae. Ideally, the effect of a change in antibiotic prophylaxis regimen should be examined in a robustly designed clinical follow-up study. A final limitation of the study is that the susceptibility of Enterobacteriaceae to cefazolin was not tested as EUCAST breakpoints for cefazolin do not exist. The susceptibility of Enterobacteriaceae to cefazolin is roughly comparable to the susceptibility to AMX and second generation cephalosporins [25], implying that cefazolin does not cover the majority of Enterobacteriaceae that caused the post-operative infections found in this study.

## Conclusions

A detailed analysis of the incidence, etiology, and timing of infections following radical cystectomy showed high incidence of post-operative infections after radical cystectomy and urinary diversion surgery. Notably, we identified a peak in bacteremia and complicated UTI immediately after stent removal. We believe that administration of antibiotic prophylaxis just before stent removal might help to reduce post-operative infections, although this needs to be confirmed in future robust clinical trials before (inter)national guidelines should be altered. Our study emphasizes that local guidelines should be adapted to local epidemiology of resistance, and that increased antimicrobial resistance necessitates a prophylactic regimen with increased gram-negative coverage in radical cystectomy.

## Additional file

Additional file 1: **Table S1.** Uni- and multivariate analysis of post-operative infections after radical cystectomy. (DOCX 15 kb)

## Abbreviations

AMC: amoxicillin-clavulanic acid
AMX: Amoxicillin
ASA: American Society of Anesthesiologists
BMI: Body mass index
CAZ: Ceftazidim
CCI: Charlson Comorbidity Index
CIP: Ciprofloxacin
CN: Gentamicin
CRO: Ceftriaxon
cUTI: Complicated urinary tract infection
EUCAST: European Committee on Antimicrobial susceptibility testing
IC: Ileal conduit
MALDI-TOF MS: Matrix-assisted laser desorption-ionization-time-of-flight mass spectrometry
MDRO: Multidrug resistant organisms
MEM: Meropenem
MIC: Minimal inhibitory concentration
NB: Neobladder
PTZ: Piperacillin-tazobactam.
SSI: Surgical site infection
SXT: Trimethoprim-sulfamethoxazole
UDS: Urinary diversion surgery
VAN: Vancomycin
