# Supplementary material for: Incidence and microbiology of post-operative infections after radical cystectomy and ureteral stent removal; a retrospective cohort study
Source: BMC Infect Dis. 2019 Apr 3;19:303. doi: 10.1186/s12879-019-3932-4 (PMC6448312; doi:10.1186/s12879-019-3932-4)
Supplement: Supplementary file 1 — Table S1. Uni- and multivariate analysis of post-operative infections after radical cystectomy. (DOCX 15 kb) [file 12879_2019_3932_MOESM1_ESM.docx]

**Additional file 1: Table S1.** Uni- and multivariate analysis of post-operative infections after radical cystectomy

| Variable | Univariate analysis p-value | Multivariate analysis p-value | Odds ratio in multivariate analysis |
| --- | --- | --- | --- |
| Sex | 0.97 |  |  |
| Age | 0.47 |  |  |
| BMI | 0.75 |  |  |
| (Previous) smoker | 0.61 |  |  |
| Charlson comorbidity index | 0.70 |  |  |
| ASA class | 0.64 |  |  |
| Colonization with MDRO | 0.07 | 0.08 | 7.1 (95% CI 0.81-62.7) |
| Optimal antibiotic prophylaxis | 0.56 |  |  |
| Type of diversion, NB versus IC | 0.03 | 0.03 | 4.1 (95% CI 1.6-10.5) |
| Duration of surgery | 0.64 |  |  |
| Blood loss | 0.47 |  |  |

Abbreviations: ASA, American Society of Anesthesiologists; BMI, body mass index; CI, confidence interval; IC, ileal conduit; MDRO, multidrug resistant microorganism; NB, Hautmann Neobladder
